# Dissecting the Interplay Mechanism between Epigenetics and Gut Microbiota: Health Maintenance and Disease Prevention

**DOI:** 10.3390/ijms22136933

**Published:** 2021-06-28

**Authors:** Yuqi Wu, Chong-Zhi Wang, Jin-Yi Wan, Haiqiang Yao, Chun-Su Yuan

**Affiliations:** 1 School of Traditional Chinese Medicine, Beijing University of Chinese Medicine, Beijing 100029, China; wyq0729@bucm.edu.cn; 2 National Institute of TCM Constitution and Preventive Medicine, Beijing University of Chinese Medicine, Beijing 100029, China; 3 Tang Center for Herbal Medicine Research, The University of Chicago, Chicago, IL 60637, USA; czwang@dacc.uchicago.edu (C.-Z.W.); cyuan@dacc.uchicago.edu (C.-S.Y.); 4 Department of Anesthesia and Critical Care, The University of Chicago, Chicago, IL 60637, USA

**Keywords:** gut microbiota, SCFAs, epigenetics, microRNA, interplay mechanism

## Abstract

The gut microbiota exists throughout the full life cycle of the human body, and it has been proven to have extensive impacts on health and disease. Accumulating evidence demonstrates that the interplay between gut microbiota and host epigenetics plays a multifaceted role in health maintenance and disease prevention. Intestinal microflora, along with their metabolites, could regulate multiple epigenetic pathways; e.g., DNA methylation, miRNA, or histone modification. Moreover, epigenetic factors can serve as mediators to coordinate gut microbiota within the host. Aiming to dissect this interplay mechanism, the present review summarizes the research profile of gut microbiota and epigenetics in detail, and further interprets the biofunctions of this interplay, especially the regulation of intestinal inflammation, the improvement of metabolic disturbances, and the inhibition of colitis events. This review provides new insights into the interplay of epigenetics and gut microbiota, and attempts to reveal the mysteries of health maintenance and disease prevention from this new perspective.

## 1. Introduction

Microbiota are ecological communities of commensal microorganisms that typically inhabit a particular environment. The human microbiota, especially gut microbiota, affect host physiology and pathology to a great extent [1]. Human beings and microbiota are engaged in a dynamic equilibrium of interdependence and interplay. Microbes are distributed on the surface and in the external cavity of the human body, such as the oral cavity, the nasal cavity, and the intestinal lumen. The largest microbial community of microbiota in the human body is located in the large intestine; these obligate anaerobic organisms are known as gut microbiota [2]. Trillions of microorganisms live in the enteric canal, forming a complex symbiotic ecosystem with the host [3,4]. It is still unclear whether gut microbiota begin to colonize the human body before or after birth [5,6].

It should be noted that gut microbiota comprehensively affect a variety of physiological and pathological processes in the human body. According to the studies we could access to date, commensal gut microbiota play a vital role in maintaining the body’s homeostasis and the occurrence of disease [7]. Microorganisms in the gut are involved in many human physiological functions; e.g., fermentation of indigestible food components and synthesis of vitamins, resistance to viruses, maintenance of intestinal homeostasis, promotion of immune system maturation, and maintenance of intestinal epithelial barrier function [8]. Therefore, gut microbiota have even been considered to be an essential organ and called “the second genome”.

In recent years, with the rapid advance of gut microbiota research, it has been revealed that acquired factors such as diet, antibiotic use, infections, etc., could regulate microbial communities via epigenetic approaches, affecting the homeostasis and disorder status of a host [9]. Epigenetic modifications can affect a host’s pathological conditions by manufacturing a nutritional profile or shaping the structure of gut microbiota. For example, microRNA (miRNAs), as a minimal mediator, can enter the gut microbiota and shape their composition, ultimately affecting the host’s intestinal health [8,10,11,12,13,14].

**Figure 1:**
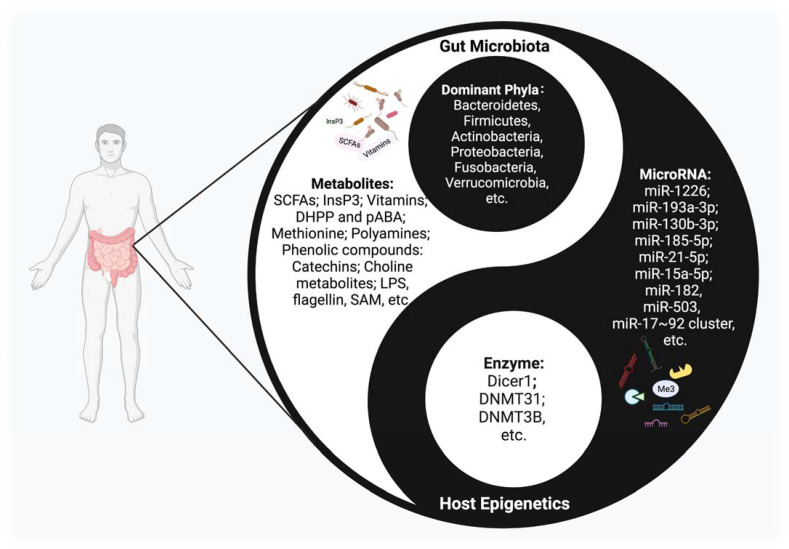
The Interplay between host epigenetic mechanisms and gut microbiota. Gut microbiota inhabit the intestinal tract and are in symbiosis harmoniously with the human body, constituting a microecology in a yin-yang dynamic balance. Once this balance is disrupted, various diseases may occur. The host regulates the diversity and composition of gut microbiota through miRNAs and other epigenetic factors. Intestinal flora, along with their metabolites, could regulate multiple epigenetic pathways; e.g., DNA methylation, miRNA, or histone modification in the host.

The crosstalk between microbiota and epigenetics, a remarkable academic focal point, has been attracting more and more attention. Various important but obscure mechanisms are thought to have been uncovered from this perspective. Studies have shown that this interplay mechanism accompanies the entire life cycle of the human body, from the fetal period to the end of life, and has an important impact on health maintenance and disease prevention [8] (Figure 1). However, at present, the exact mechanism of the interplay between gut microbiota and epigenetics is only the tip of the iceberg.

With the development of advanced computational tools for metagenomics analyses based on whole-genome sequencing and other techniques, many enlightening studies in this field could promote our understanding of the crosstalk between gut microbiota and epigenetics [15,16]. The regulation of gut microbiota by the host can affect the response to physiological stimuli and the process of disease development, which is a promising new field because of its potential biomedical significance. Aiming to dissect this interplay mechanism, the present review will interpret the research profile of gut microbiota and epigenetics in detail. Moreover, we focus on the interplay between these two factors and further biofunctions, including regulation of intestinal inflammation, improvement of metabolic disturbances, and inhibition of colitis events. This review provides new insights into the interplay of epigenetics and gut microbiota, and attempts to reveal the mysteries of health maintenance and disease prevention from this new perspective.

## 2. Overviews of Epigenetics and Gut Microbiota Research

### 2.1. Epigenetics

Epigenetics is the study of changes in organisms caused by the modification of gene expression rather than the alteration of a DNA sequence; meanwhile, phenotype changes can be stably transmitted in the process of growth and development [17,18]. Epigenetic mechanisms include DNA methylation, histone modification, and noncoding RNAs. These host epigenetic modifications play an intermediary role in intestinal homeostasis, inflammatory regulation, and metabolic disorders, and provide a basis for maintaining the intestinal commensal system.

#### 2.1.1. DNA Methylation

DNA methylation is an important epigenetic regulatory mechanism in the human body that affects gene expression by regulating the accessibility of transcription factors [19,20]. The mechanism that allows DNA methylation sites to be retained during DNA replication is characteristic of epigenetic systems, and is a process that benefits from specific proteins that recognize CpG hemimethylation in DNA, thereby reproducing DNA methylation on newly replicated DNA [21]. DNA methylation is operated by the DNA methyltransferase DNMT1 and its assistant UHRF1 (also known as NP95), which specifically binds hemimethylated DNA by stimulating DNMT1 via its ubiquitin ligase. Therefore, gene complexes are tightly linked to the “author” and “reader” of epigenetic methyl CpG marks, two roles that are essential for maintaining DNA methylation [22]. This process needs numerous raw materials; dietary-derived nutrients such as folate and vitamin B12 could serve as methyl donors, and methyl groups catalyzed by DNA methyltransferase can be transferred to the basic group in the DNA sequence [23]. DNMT can add a methyl group from the donor S-adenosylmethionine (SAM) to the carbon-5 position of the cytosine (5mC), while the ten-eleven translocation enzyme (TET) dioxygenase family can actively reverse this process by oxidizing 5mC to 5-hydroxy-methylcytosine (5hmC) [19]. It is generally believed that there are four kinds of DNMT in mammals, and these can be divided into two categories: DNMT1 and DNMT3. DNMT1 maintains its methylation during DNA replication and repair, and DNMT3 catalyzes the de novo methylation of CPG [24]. Comparing the gut microbiota of GF and conventional mice, the degree of intestinal DNA methylation was significantly reduced in the absence of gut microbiota [25]. This hypomethylation is not caused by a low level of DNMT activity; however, the underlying reason is supposed to be the reduction of one-carbon metabolites derived from gut microbiota [26].

#### 2.1.2. Histone Modification

Histones are important components of chromatin, and they fold DNA to assemble nucleosome structures consisting of H2A, H2B, H3, and H4. Histone modification, as an important epigenetic pathway, plays an important role in regulating gene replication, transcription, and DNA damage repair. Multiple modifications can be made to histones, including acetylation, methylation, phosphorylation, and ubiquitination, primarily in the N-terminal histone tails, and most of the histone modifications are reversible and responsive to metabolic changes [20].

Histone acetylation, an important type of histone modification, is generally related to active gene transcription, while deacetylation is related to the inhibition of transcription [20]. The status of histone acetylation is regulated by both histone acetyltransferases (HATs) and histone deacetylases (HDACs), which play opposite roles. The biofunction of HATs is to catalyze the deposition of acetyl group by transferring an acetyl group from acetyl-coenzyme A to the ε-amino group of lysine residues. Histone acetylation can also expose the target sites of transcription factors in nucleosome DNA and initiate the assembly of transcription complexes. Contrary to the functions of HATs, HDACs catalyze deacetylation to erase the acetyl group from the tail of histone leading to a reduction of accessibility.

Histone methylation is catalyzed by a large number of histone methyltransferases. There are multiple forms of histone methylation, depending on which amino acids in the histones are methylated and how many methyl groups are attached. The most common form is the methylation of lysine residue ε-amino. Histone methylation can regulate the transcription of genes, including both promotion and inhibition, under different conditions [27]. Histone methyltransferases (HMT) have different specificities for histone substrates, but all known HMTs use S-adenosylmethionine (SAM, also known as AdoMet) as a methyl donor and produce S-adenosylhomocysteine (SAH, also known as AdoHcy) as a byproduct. SAH is a competitive inhibitor of SAM and a noncompetitive or mixed peptide substrate inhibitor [27].

There are several ways in which gut microbiota can regulate histone modifications. Generating numerous bioactive compounds from microbial metabolism is one effective mechanism. Acetate and propionate can inhibit HDAC2 and HDAC3, and butyrate can inhibit the activity of HDAC1 and HDAC2, thereby affecting the stability of intestinal ecology [28,29]. Meanwhile, histone methylation or demethylation can be globally modulated by various cellular metabolites/cofactors, including SAM, Fe^2+^/Fe^3+^, and α-KG [30].

#### 2.1.3. Noncoding RNA

The discovery of noncoding RNAs is considered a scientific breakthrough in the “genetic central dogma” (DNA→RNA→Protein) and the “junk RNA” theory, in which the junk RNA contains all those RNAs that are not translated—about 70% of the genome is transcribed, while about only 2% is translated—and, therefore, they are considered non-functional [31]. Noncoding RNAs participate in important biological processes, but do not encode proteins. Thus far, these molecules have been widely demonstrated to play a role in the regulation of the translation and transcription of coding and noncoding genes. Based on their sizes, regulatory noncoding RNAs can be divided into long ncRNAs (lncRNAs; >200–300 bp) and small ncRNAs (<200–300 bp), including miRNAs; short interfering RNAs (siRNAs); endo-siRNAs; and PIWI-interacting RNAs (piRNAs) [32]. Noncoding RNAs have many important functions, including (1) structural functions, such as the construction of ribosomal subunits (rRNAs); (2) transport functions during protein translation, in which tRNAs interact with RNA ligands and mRNAs; and (3) regulatory functions by regulating RNA, DNA, and proteins [33].

In particular, lncRNAs have been reported to be useful for distinguishing types of gut microbiota and as biomarkers for identifying the process of host–microbiota interplay [34]. The expression of lncRNAs in mice with or without the presence of microflora are revealed to be distinct. Furthermore, the different strains of microbes may also affect the lncRNA expression. Mice inoculated with wild-type *E. coli* and *E. coli* expressing bile salts hydrolase induced specific changes of lncRNA profiles [34].

MiRNA, an endogenous small noncoding RNA molecule, contains about 18 to 25 nucleotides and can regulate gene expression by inducing degradation of mRNAs or inhibiting translation. Meanwhile, miRNA can regulate various physiological or pathological pathways such as cell differentiation, cell proliferation, and tumor development; approximately 30% of protein-encoding genes are regulated by miRNAs [35]. MiRNAs also can regulate the commensal microbiota-dependent intestinal epithelial cells that maintain gut homeostasis and dysbiosis [36]. Meanwhile, miRNA is an important regulator of the immune pathway that is involved in homeostasis. The dysfunction of miRNAs causes a wide variety of body abnormalities, including cancer and autoimmune diseases [37].

### 2.2. Gut Microbiota

In human gut microbiota, the dominant phyla are *Bacteroidetes*, *Firmicutes*, *Actinobacteria*, *Proteobacteria*, *Fusobacteria*, and *Verrucomicrobia*, two of which—*Firmicutes* and *Bacteroidetes*—account for 90% of gut microbiota [2,38]. Although mature microbiota are rather stable, their richness and composition may fluctuate with internal and external factors, including age, region, lifestyle, drug use, and dietary habits. Gut microbiota are present on the surface of the intestinal mucosa and vary according to the different anatomical regions of the gut; they play an essential role in digesting food, obtaining energy, and maintaining homeostasis [38,39].

In particular, gut microbiota generate numerous bioactive compounds, including short-chain fatty acids (SCFAs) and choline metabolites and lipids [39], which are important factors affecting host physiology and pathology [8]. Microbial metabolites are key messengers in the interplay between microbiota and epigenetics. They not only produce local effects in the gut, but also regulate distant organs, such as the lungs, the heart, and the brain [40]. The dysbiosis of gut microbiota can induce a series of diseases, such as obesity, diabetes, metabolic syndrome, and inflammatory bowel disease (IBD) [41,42,43,44]. The microbial community plays a key role in maintaining the balance of the intestinal environment.

Until recently, research has mainly focused on exploring the occurrence, development, and mechanism of disease from the perspective of gut microbiota. However, the interplay between intestinal microorganisms and epigenetics is receiving increased attention, and multiple studies have demonstrated the significance of this mechanism in health maintenance and disease prevention.

## 3. The Effects of Epigenetic Regulation on Gut Microbiota

### 3.1. Modulation of the Gut Microbiota Composition

Epigenetic mechanisms can influence the microbial community and microbiota-derived metabolites; specifically, miRNA is deeply involved in microbiota shaping [45]. Recent studies have shown that host miRNAs are involved in the regulation of gut microbiota. MiRNAs in the intestinal lumen are derived from intestinal epithelial cells (IECs), as well as goblet and paneth cells, and are excreted through exosomes [46]. MiRNAs from the host can specifically regulate the transcription of microbial genes and then affect the growth of intestinal microflora and microbiota structure [47]. A multitude of studies have confirmed that miRNAs can control the growth of microbiota and shape the structure of the microbial community [48,49]. For example, coculture of the miRNAs with microbiota can induce significant gene expression changes in microbiota [46]. By adding synthesized miRNA to the bacterial culture medium in vitro, we found that miRNA changed the gene expression of bacteria, such as *F. nucleatum* and *E. coli.* Furthermore, mice given specific miRNA also demonstrated changes in the *E. coli* growth and bacterial gene transcripts. The results indicated the levels of miRNA were negatively correlated with the abundance of the gut microbiota [47] (Table 1).

### 3.2. Adjustment of the Intestinal Homeostasis

Host epigenetics, especially miRNAs, can participate in the physiological functions related to maintaining intestinal homeostasis by regulating gut microbiota. For example, the expression of miR-21-5p in IECs can regulate intestinal epithelial permeability through ADP ribosylation factor 4 (ARF4) [50]. Studies found that the loss of host miRNAs in feces, or the selective knockout of miRNA-processing enzymes in intestinal epithelium, may lead to an imbalance of microbial homeostasis and an aggravation of colitis. These phenomena demonstrate that host-secreted miRNAs could send feedback to the gut microbiota to maintain intestinal homeostasis.

Gut microbiota, along with the intestinal epithelium and mucosal system, establish a complex intestinal barrier against enteropathogens [51]. A variety of epigenetic mechanisms are involved in the process of intestinal barrier formation through regulating the differentiation of IECs, as well as the richness of gut microbiota and their derived metabolites [52]. Host epigenetic effects vary between different types of gut microbiota. Related studies suggest that host-secreted miRNAs have an important impact on intestinal homeostasis by regulating the growth and structure of microbial communities, providing a new perspective for maintaining intestinal health [48]. However, the detailed mechanism of how these epigenetic factors interplay with different cues from symbiotic microbiota still appears foggy, and further investigation into this topic is needed in the future.

### 3.3. Regulation of the Host Metabolism

**Figure 2:**
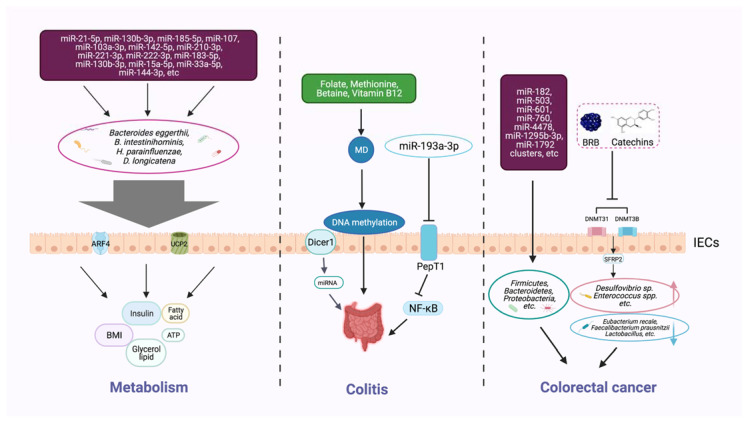
Host epigenetic factors regulate the gut microbiota in multiple progresses of diseases. The gut epithelium secretes a variety of miRNAs, which can enter microorganisms to affect their transcription and alter the microbial structure and diversity. The gut microbiota generates different metabolites (e.g., butyrate and bile acids) that can, in turn, regulate the host metabolism, including BMI, insulin secretion, and lipid production. Dietary intake of nutrients (e.g., folic acid, methionine, and vitamin B12) provides methyl donors that affect host DNA methylation, which may modulate the intestinal inflammatory state. Some microbial metabolites can modify DNA methylation, histone acetylation, and miRNAs, and impair the homeostasis of the intestinal environment, thereby reducing beneficial microbiota and increasing the richness of pathogenic bacteria, and inducing the development of colorectal cancer.

Gut microbiota and their metabolites have been identified as effective metabolic regulators, and epigenetic mechanisms could influence the host metabolism via this pathway [48] (Figure 2). The latest studies show that the interplay between circulating miRNA and gut microbiota serves as a notable mechanism in obesity [53]. Compared with the control group, 26 different circulating miRNAs and 12 microbial species were screened in an obese group, and these target miRNAs and microbes were significantly related. Three miRNAs (miR-130b-3p, miR-185-5p, and miR-21-5p) showed a negative correlation with *Bacteroides eggerthii* and exerted a BMI-regulating function. Furthermore, the expressions of miR-107, miR-103a-3p, miR-222-3p, and miR-142-5p were negatively associated with *B. intestinihominis* abundance. These miRNAs regulate genes involved in metabolism-related pathways, including fatty-acid degradation, insulin signaling, and glycerol lipid metabolism. MiR-15a promotes insulin biosynthesis by inhibiting the expression of endogenous uncoupling protein 2 (UCP2), increasing the level of ATP and glucose-stimulated insulin secretion. The expression of miR-15a-5p was negatively correlated with the abundance of *H. parainfluenzae* and could affect insulin levels, as was demonstrated by in vivo data. Meanwhile, a bioinformatic analysis showed that the expression levels of 14 circulating miRNAs (miR-107, miR-103a-3p, miR-142-5p, miR-222-3p, miR-221-3p, miR-183-5p, miR-130b-3p, miR-15a-5p, miR-33a-5p, miR-210-3p, miR-144-3p, miR-185-5p, miR-130a-3p, and miR-21-5p) and the richness of four intestinal microflora taxa (*D. longicatena, B. intestinihominis, B. eggerthii,* and *H. parainfluenzae*) in the obese group were significantly different; a correlation analysis indicated that there is an interplay mechanism between miRNAs and microbes [53]. The result indicated that miRNAs play a role in communication between the gut microbiota and the host, and that the crosstalk between epigenetics and gut microbiota is a potential target for treating metabolic disorders; however, further investigation is needed to uncover the mechanism more deeply.

### 3.4. Factors Involved in the Progress of Colitis

Epigenetic factors that arise through manipulating gut microbiota can affect the occurrence and development of colitis (Figure 2). Epigenetic-related “writer” and “eraser” enzymes play a crucial role in preventing IBD. For example, when intestinal epithelial cells lack the enzyme Dicer1, which is essential for miRNA processing, the expression level of miRNA in gut contents and feces will be reduced, resulting in gut microbiota imbalance and severe colitis [46]. Furthermore, fecal miRNA transplantation recovers the structure of gut microbiota and improves IBD. These findings indicate that miRNA plays a physiological role in shaping gut microbiota and improving intestinal inflammation. Another study found that miRNA-193a-3p indirectly reduces microbiota-metabolite-induced colon inflammation. PepT1 is a transporter that can absorb microbial products. *PepT1* was found to be upregulated and negatively correlated with mir-193a-3p in ulcerative colitis (UC). Further verification showed that mir-193a-3p decreased the activity and expression of target gene *PepT1*, and subsequently reduced the uptake of microbial products and inhibited the NF-κB pathway. These results suggest that miR-193a-3p can mitigate the intestinal inflammation caused by microbial products and protect intestinal homeostasis [54] (Table 1).

**Table 1:** Mechanisms of epigenetic modifications that regulate gut microbiota.

Epigenetic Factors	Epigenetic-Associated Gut Microbiota	Mechanism	Effects on Health or Diseases	Reference
MicroRNA-1226	*Escherichia coli*	Enter the interior of strains	Regulate microbial gene transcripts and affect microbial growth	[47]
MicroRNA-193a-3p	Gut microbiota metabolism	Downregulate PepT1 and then suppress the NF-κB pathway by reducing the intake of gut microbiota metabolism in the colon	Reduce inflammatory in the colon, protect gut homeostasis	[54]
MiR-130b-3p, MiR-185-5p, MiR-21-5p	*Bacteroides eggerthi*	Negatively regulate the expression of genes	BMI and host metabolism pathways (fatty-acid degradation, insulin signaling, glycerol lipid metabolism)	[53]
MicroR-15a-5p	*H. parainfluenzae*	Negative correlation	Inhibit the expression of endogenous UCP2; increase the level of ATP, and stimulate insulin secretion	[53]
MicroRNA enzyme Dicer1	Structure of gut microbiota	Reduce the level of miRNA	The absence of Dicer1 may result in dysbiosis of gut microbiota and aggravation of colitis	[46]
76 microRNAs (including miR-182, miR-503, and miR-17~92 cluster, etc.)	*Firmicutes*, *Bacteroidetes*, and *Proteobacteria*	A mediating role in which they transfer to stromal cells and immune cells if miRNAs in tumor cells are out of balance	Affect tumor metastasis and invasion	[55]
DNMT31 and DNMT3B promote the methylation of SFRP2 promoter	*Desulfovibrio* sp. and *Enterococcus* spp.; *Eubacterium rectale, Faecalibacterium prausnitzii and Lactobacillus*	Decrease of *Desulfovibrio* sp. and *Enterococcus* spp.; Increase of *Eubacterium rectale*, *Faecalibacterium prausnitzii,* and *Lactobacillus*	Increase intestinal inflammation and risk of colon cancer	[56]

Methyl-donors (MD), including many dietary supplements, such as folate, methionine, betaine, and vitamin B12, can provide essential raw material in DNA methylation processes [57]. The colon is an important environment for the folate metabolic cycle to maintain local homeostasis [58]. In a mouse model of Crohn’s disease (CD), methyl-donor supplementation can prevent the intestinal colonization of adherent-invasive *E. coli,* showing beneficial effects on the inflammation. Furthermore, the serum folate concentration was found to be inversely correlated to fecal inflammatory markers in a cohort of CD [59]. MD supplementation during pregnancy increased the odds of colitis infection in offspring, and was found to be associated with persistent epigenetic and intestinal microbial changes [57]. Methionine is an active MD, and short-term dietary methionine supplementation in a mouse model may alter the DNA methylation in the gut; regulate the microbiome profiles; and affect the intestinal barrier function, gene expression, and histomorphology [60].

### 3.5. Factors Associated with Colorectal Cancer

Epigenetic changes in the colon can markedly help us identify and prevent intestinal tumors (Figure 2). For instance, changes in miRNAs have been observed to affect the state of gut tumor metastasis and invasion. Accumulating evidence confirmed that miRNAs are involved in the occurrence, development, and response to treatment of tumors, and can be used as biomarkers for diagnosis, prognosis, and prediction of therapeutic effects [50,61]. Compared with healthy controls, the plasma levels of miR-601 and miR-760 in colorectal cancer (CRC) patients were significantly lower, which is helpful for the early diagnosis of CRC [62]. Moreover, lower expression levels of mir-4478 and mir-1295b-3p in fecal samples could also serve as potential noninvasive molecular markers for CRC diagnosis [63].

Further investigation revealed that the specific miRNAs in CRC are closely linked with microorganisms [55]. A total of 76 miRNAs, including the known miR-182, miR-503, and miR-1792 clusters, were differently expressed in tumor tissues than in healthy tissues in CRC; those miRNAs were associated with the presence of several phyla of microbes, such as Firmicutes, Bacteroidetes, and Proteobacteria. The imbalance of miRNA in tumor cells may affect the survival of gut microbiota or regulate the gene expression of certain microbes [55] (Table 1). Studies have confirmed that the microbial community relies on miRNAs that play a mediating role in maintaining crosstalk with the host [64,65].

Microarray analysis identified genes with different DNA methylation in colon cancer [66]. According to another study, black raspberry BRB anthocyanins could downregulate the expression of DNMT3A, DNMT3B, and p-STAT3 in CRC, resulting in the demethylation of the SFRP2 gene promoter and increasing the expression of SFRP2 at both the mRNA and protein levels. Along with this epigenetic change, the microbial structure has also been modified, which implies an inner correlation of DNA methylation and gut microbiota. The interplay of the two factors is supposed to play a crucial role in the prevention of CRC [56].

## 4. The Effects of Gut Microbiota on Epigenetic Factors

### 4.1. Inducement of Host Epigenetic Changes

As an indispensable symbiont of the human body, microbiota can induce epigenetic modifications in a host. More specifically, the human body can adapt to environmental stimuli via DNA methylation, histone modifications, or other epigenetic approaches [67]. Gut microbiota regulate host epigenetics, primarily by producing metabolites to maintain the dynamic balance of the body, such as generating SCFAs to modify the host epigenome, which affects the body’s state of health and its diseases [68,69,70,71].

Gut microbiota can synthesize biological compounds to provide raw materials; e.g., methyl or acetyl groups for DNA methylation or histone modification, which may affect the physiological and pathological processes of host epigenetics [72,73]. In previous research, DNA methylation and gene expression were examined in the colonic mucosa of Toll-like receptor 2 (*TLR2*) knockout mice. In this *TLR2^−/−^* mouse model, two genes involved in immune processes, *Anpep* (alanyl aminopeptidase, membrane) and *IFIT2* (interferon-induced tetrapeptide repeat 2 protein), were hypermethylated in the promoter region. Epigenomic and transcriptomic modifications are associated with alterations in mucosal microbial composition. Several microbial species, including members of the Firmicutes genus, showed a significant difference in abundance between wild-type and *TLR2^−/−^* animals. These results suggest that alterations in mucosal microbial composition caused by *TLR2* deletion may lead to alterations in epigenetic modification [74].

Gut microbiota can be linked with host epigenetics through the use of biomolecules produced by metabolizing the host’s diet, including SCFAs, polyphenols, vitamins, tryptophan catabolites, and polyamines [75]. However, the deep molecular mechanism underlying this interplay has not been elucidated thoroughly. Studies found that SCFAs, some of the most important mediators, participate in this interplay process. The rapid increase or decrease in SCFAs induced by dietary nutrient intake or environmental changes can cause eventual epigenetic modifications in the host. For example, butyrate can promote intestinal proliferation and maintain homeostasis [76,77], and it functions through different signaling pathways [77,78,79]. Moreover, gut microbiota can modify host-cell responses to stimuli by altering host epigenetics to control gene expression [8].

Both the gut microbiota (Table 2) and microbiota-derived metabolites (Table 3) can induce specific epigenetic changes to regulate multiple physiological functions of the host. For instance, SCFAs derived by microbial metabolism are important energy sources for gut microbiota and host intestinal epithelial cells [13,80]. An important function of SCFAs is to adjust the host’s homeostasis by regulating epigenetic pathways [81,82,83,84]. The most abundant SCFAs in the colon are acetate and propionate. Propionate is mainly produced through the succinate pathway by Bacteroides and many Negativicutes (such as *Veilonella*, *Roseburia*, and *Ruminococcus*) [85]. Butyrate can induce the differentiation of colonic Treg cells in mice and enhance histone H3 acetylation in the promoter and conserved noncoding sequence regions of the Foxp3 locus [86]. As an indispensable energy source of IECs, butyrate can be generated from butyryl-CoA through the acetate CoA-transferase pathway by the phylum Firmicutes [80,87,88]. Propionate and butyrate can also be obtained from peptides and the fermentation of amino acids. Both can inhibit the activity of HDACs in IECs and immune cells by promoting the hyperacetylation of histones and certain transcription factors involved in signal transduction; they therefore play a vital role in cancer development [89]. Acetate and propionate are absorbed by colon cells through passive diffusion, electroneutralization, or current absorption, and are then transported in peripheral tissues [90]. It has been experimentally proven that concentrations of SCFAs in the intestinal content of germ-free mice are markedly lower than in conventional mice [90]. Meanwhile, it is notable that the concentrations of SCFAs in feces could not reflect the rate of production of SCFAs in the gut lumen, as most SCFAs could be absorbed by the host [91]. Some derivative aromatic SCFAs, i.e., phenylacetate and phenylbutyrate could be generated by the biotransformation certain microbial species, such as *Bacteroides, Clostridium, Eubacterium limosum,* and *Eggerthella lenta* [75].

**Table 2:** Mechanisms of gut microbiota that induce host epigenetic changes.

Gut Microbiota	Microbiota-Associated Epigenetic Changes	Mechanism	Effects on Health or Diseases	Reference
Gut microbiota	DNA methylation	Possibly activate DNMT1 through metabolites, then regulate the methylation of 3 ‘CpG islands (CGIS)	A benefit to the maturation of epithelial cells	[92]
	Histone H3 acetylation	Enhance histone H3 acetylation in the promoter and conserve noncoding sequence regions of the Foxp3 locus	Nm	[86]
	HDAC3	Program diurnal metabolic rhythms, coactivate ERRα transcription of the lipid transporter gene CD36, and promote lipid absorption and dietary-induced obesity	Induce microbiota-dependent rhythmic	[93]
	MicroR-107	Affect the activity of the MyD88 and NF-κB pathways; target IL-23p19 gene expression	Treatment of IBD and maintenance of host gut homeostasis	[94]
*Mycobacterium tuberculosis, Helicobacter pylori and Salmonella enterica*	MiR-let-7f	Mycobacterium tuberculosis downregulates miR-let-7f by secreting ESAT-6. Mir-let-7f targets TNFAIP3, which is a negative regulator of the NF-κB pathway	Activate host immune response and reduce microbial survival	[95,96]
*Mycobacterium tuberculosis*	DNA demethylation	Induce demethylation by oxidating 5mC to 5hmC via TET family proteins	Immune transcription factors, active histone marker sites, and increase chromatin accessibility	[97]
*Akkermansia muciniphila* and *Lactobacillus plantarum*	Modification of N6-methyladenosine (m^6^A)	Expression of Mettl16 and its target mRNA encoded S-adenosylmethionine synthase Mat2a methylation	Affect the metabolism, inflammation, and antibacterial process of the host	[98]
*Lactobacilli*	Downregulate miRNAs (miR-200b, miR-215, and miR-192)	Nm	Maintain homeostasis and shape the host response to infection	[99]
*Leuconostoc mesenteroides*	MiRNA-21, miRNA-200b	Nm	Promote the apoptosis of colon cancer cells	[100]
*Listeria monocytogenes*	Histone H3, histone H4, IL8 promoter	Acetylation of histone H4 (lysine 8) and phosphorylation/acetylation of histone H3 (serine 10/lysine 14) and at the IL8 promoter in HUVEC cells as well as recruitment of the histone acetylate cyclic adenosine 3	Nm	[101]
Commensal microbiome-dependent (*Bacteroides acidifaciens* type A43 (BA) and *Lactobacillus johnsonii* 129 (LJ), etc.)	MiR-21-5p	Commensal microbiome-dependent miR-21-5p expression in IECs regulates intestinal epithelial permeability via ARF4	Regulate intestinal epithelial permeability	[50]
*Fusobacterium nucleatum*	Toll-like receptor, miRNAs (miR-4802 and miR-18a *, etc.),	Downregulate miR-4802 and miR-18a *, leading to depressurization of the autophagy proteins ATG7 and ULK1, respectively	Improve the response to chemotherapy, reduce cancer recurrence	[102]

Nm: not mentioned.

**Table 3:** Mechanisms of metabolites derived by gut microbiota to mediate host epigenetic changes.

Gut Microbiota Metabolites	Metabolites-Associated Epigenetic Changes	Epigenetic-Associated Mechanism	Effects on Health or Diseases	Reference
SCFAs	HDACs, GPCRs	Inhibit HDACs and increase FOXP3 protein acetylation and gene expression in CD4+T cells, promote Treg cells differentiation in the extrathymic, and promote the expression of β-defensin-2 and β-defensin-3, GPCRs; suppress the activation of NF-κB and STAT1	Exert anti-inflammatory activity	[103,104,105,106,107,108]
	HDAC3	Coactivate ERRα	Program diurnal metabolic rhythms; promote lipid absorption and dietary-induced obesity	[93]
	① GPR43(FFAR2); ② H3K27me3 and H3K4me3 histone	① Inhibit cAMP-PKA-CREB pathway activity that leads to the overexpression of HDACs, ② Link to the promoter regions of inflammation suppressors (*sfrp1, dkk3, socs1*, etc.)	Regulate colonic inflammation, protect against colon carcinogenesis	[109]
	SLC5A8	As a plasma membrane transporter of SCFAs, SLC5A8 promotes butyrate to enter cells and inhibit HDACs	Apoptosis in cancer cells	[110,111]
	Nuclear SIRT1	Increase its availability from precursors and by producing resveratrol derivatives	Regulation of mitochondrial biogenesis, metabolism, stress responses, genome stability, and ultimately aging	[112,113]
	Activate Stat3 and inhibit HDACs	Inhibit claudin-2, IL-10RA dependent mechanism	Promote the formation of epithelial barrier	[114]
Butyrate	Inhibit HDAC1 and HDAC2	Induce histone hyperacetylation and gene transcription	Inhibit cell proliferation, promote differentiation, treat cancer	[29,76]
	Inhibit HDAC3	① Induce the antibacterial activity of intestinal macrophages; ② Decrease activity of HDAC3 in IECs	① Enhance the intestinal resistance to pathogens; ② Prevent diet-induced obesity	[115,116]
	Downregulate miR-24	Downregulate XIAP expression to resist caspase inhibition	Apoptosis in cancer cells	[79,117]
	FFAR3 and LINE1 gene hypomethylation	DNA methylation of FFAR3 and LINE1 gene	Affect metabolic diseases (obesity and type 2 diabetes)	[118]
	H3K27me3	H3K27me3 enrichment is negatively correlated with the concentration-dependent downregulation of NFκB1	H3K27me3 of NFκB1 promoter in colon tissue is increased, then reducing intestinal inflammation	[119]
	Reduce the levels of the miR-17-92a (oncomiR-1)	Butyrate inhibits miR-92a transcription by reducing the expression of c-Myc protein, which is mediated by the interplay between C12*orf*25 promoter and c-Myc, thus augmenting p57 levels	Reduce the proliferation and apoptosis of colon cancer cells; stimulate apoptosis	[120]
Acetate and propionate	HDAC2 and HDAC3	Inhibit HDAC2 and HDAC3	Nm	[29]
Butyrate and propionate	Inhibit HDAC	Downregulate *Aicda* and *Prdm1* mRNA-3’UTRs, then reduce *Aicda* and *Prdm1* in B cells	Inhibit the production of autoantibodies and autoimmunity in mice with lupus erythematosus	[121]
Inositol-1,4,5-trisphosphate (InsP3)	Activate HDAC3	Antagonistic effect of butyrate on HDAC3	Activate histone deacetylase in IECs and promote epithelial repair	[52]
Vitamins, DHPP and pABA	DNMTs and HMTs	Generate substrate SAM, which acts as methyl-donating for DNMTs and HMTs	Nm	[58,122]
Methionine	DNA methylation	Generate substrates for SAM synthesis	Formation of host microbiota; regulate microbiota metabolism	[123,124]
Polyamines (arginine, spermidine, putrescine)	DNA methylation; DNMT	Reverse the overall aberrations of DNA methylation; inhibit DNMT activity by increasing dcAdoMet	Reverse aging; potentially treat cancer and metabolic disorders	[125,126,127]
Phenolic compounds: catechins	Increase DNMT1 in the colon	Inhibit the activity of DNMTs via degradation of catechins to produce phenolic acids	Inhibit tumor tissue growth	[128,129]
Choline metabolites	Act as methyl donors for DNA methylation	Affect the level of methyl donor metabolites in plasma and liver	Change the composition of microbiota and affect the susceptibility to colitis and metabolic diseases	[57,130]
LPS	Methylation of *TLR4*	Decrease transcriptional activity at this locus and lead to decreased reactions to LPS	Activate the innate immune system	[131]

Nm: not mentioned.

### 4.2. Modulation of Intestinal Homeostasis

DNA Methylation: The epithelial cells on the surface of the intestinal cavity have been exposed to symbiotic microbiota for a long time. IECs not only physically separate the contents of the intestinal cavity from the internal environment, but also actively participate in the immune response as the front-line defense of the mucosal immune system. Studies have shown that IECs are stimulated by symbionts through Toll-like receptors (TLRs) to maintain their homeostasis. The hyporesponsiveness of human IECs to lipopolysaccharide (LPS) is due to the downregulation of gene *TLR4*, which is mediated by epigenetic mechanisms [131]. In adult IECs, the DNA methylation of gene *TLR4* depends on gut commensals, which can generate signaling molecules to modulate significant inflammatory responses against diseases [10,131]. The *TLR4* is a member of the Toll-like receptor family that recognizes LPS and activates the innate immune system. The commensal gut microbiota were proved to increase the methylation level in the *TLR4*, thereby downregulating its transcriptional activity and leading to a decreased reaction to LPS by maintaining the insensitivity of the microbiota within the colon [131].

Another study also confirmed the impact of gut microbiota on the regulation of host DNA methylation. After birth, the development of intestinal stem cells is of great significance to maintaining lifelong health in the gut. This study found that *Enterobacteria* appeared in the first 3–14 months of life, and more precociously in natural breastfeeding, which will itself end after the third or fourth year [132]. The epigenetic inheritance of intestinal stem cells requires DNA methylation, but this process is controlled by the gut microbiota: gut microbiota could activate DNMT1 and regulate the methylation of 3’CpG islands (CGIS) that contribute to the maturation of epithelial cells [92] (Table 2).

Gut microbiota also affect the markers of the host epigenetic transcriptome. Compared with normal, modified, and germ-free (GF) mice, the changes in gut microbiota were related to the modification of N6-methyladenosine (m^6^A) in the cecum and decreased expression in the liver, which affected the metabolism, inflammation, and antibacterial process of the host. By analyzing the expression of several known enzymes, it became clear that the absence of microbiota may result in decreased expression levels of Mettl16 and the hypomethylation of *Mat2a*. In addition, *Akkermansia muciniphila* and *Lactobacillus plantarum* could influence the modification of m^6^A in mono-associated mice [98].

Gut microbiota can synthesize biological compounds, including folate and vitamins B2, folate (B9), and B12, which are related to DNA methylation [133]. Meanwhile, dietary methionine is not only involved in the formation of the microbial community [123], as it also regulates microbiota metabolism to generate substrates for S-adenosylmethionine (SAM) synthesis, which is required for DNA methylation [122,124]. Except for diet, specific gut microbiota can also synthesize methyl-donating folate from pteridine precursors (DHPP) and p-aminobenzoic acids (pABA), such as commensal *Lactobacillus* and *Bifidobacterium* species, which are regarded as probiotics [58]. A series of studies demonstrated that microbiota affect the methylation profile of IECs, thus playing a functional role in maintaining intestinal homeostasis. In the colonic epithelial cells of germ-free mice, a low level of folate cycle induces hyper DNA methylation, which is also related to the significant loss of ten-eleven translocation activity and lower activity of DNA methyltransferase [131].

Histone modification: As an important epigenetic regulator, HDAC can receive signals from gut microbiota and diet, and its expression in IECs is of great significance for maintaining intestinal homeostasis. The transplantation of gut microbiota into germ-free mice can effectively modify the histone code in a tissue-specific manner. For instance, levels of H3K27me1 and H3K36me2 were observed to be increased in adipose tissues while decreased in the liver and colon. The ability of specific microorganisms to activate major histocompatibility complex (MHC) class II genes has also been demonstrated by germ-free mice, and mechanisms of DNA methylation and histone acetylation may be involved in the regulation of MHC genes [134,135].

*Listeria monocytogenes*, a foodborne Gram-positive facultative anaerobe, could induce acetylation of histone H4 (lysine 8) in a time-dependent manner. It can also induce phosphorylation and acetylation of histone H3 (serine 10/lysine 14) [101] (Table 2). Furthermore, gut microbiota can affect histone modification by altering the activity of enzymes or the levels of substrates [8]. More molecular mechanisms of gut-microbiota-dependent histone modifications are starting to be elucidated. Butyrate is a metabolite of anaerobic fermentation and a famous histone deacetylase inhibitor that works by linking with the Zn^2+^ at the Sp1/SP3 binding site. Its HDAC activity is inhibited [136], resulting in histone hyperacetylation and transcriptional activation of the p21 (WAF1/CIP1) gene, which induces the production of inflammatory cytokines IL-1B and IL-8 [137,138], and also inhibits the activation of NF-κB and STAT1 in downstream pathways to exert its biofunctions [29,103]. Butyrate has been observed to increase the acetylation and decrease LPS-induced expression of the proinflammatory cytokines IL-6 and IL-12 in macrophages [139]. The administration of butyrate, acetate, propionate, and valerate in cell lines may result in an increase of acetylation in histone H3 and H4, which can further induce cell cycle arrest and the downregulation of an apoptotic and angiogenesis regulator neuropilin-1 (NRP1) [140].

SCFAs, especially butyrate, can regulate the sirtuin deacetylase SIRT1, which plays an important role in the regulation of mitochondrial biogenesis, metabolism, stress responses, genome stability, and aging [112,141]. Resveratrol has been shown, by enhancing the SIRT1 activity, to ameliorate metabolic syndrome [113] (Table 3). The beneficial effects of veratrol depend on the metabolism of gut microbiota (mainly *Bifidobacterium infantis* and *Lactobacillus acidophilus*), which can improve the bioavailability of resveratrol and increase the production of resveratrol derivatives [142].

Long noncoding RNAs: A resource of gut-microbiota-associated lncRNAs has been reported for screening lncRNA biomarkers and identifying functional lncRNAs in host–microbe interactions [34]. Gut microbiota can regulate the expression of lncRNAs in the local intestine; e.g., jejunum, and consequently regulate the tissue-specific pathway, together with adjacent protein-coding genes [143]; however, further validation experiments should be conducted to attain more solid conclusions.

MiRNAs: The pretreatment of *lactobacilli* may counteract the down-regulation of miRNAs (miR-200b, miR-215, and miR-192) typically induced by *L. monocytogenes* infection. Probiotics that regulate the expression of miRNA could exert beneficial effects on the maintenance of intestinal homeostasis and the shaping of the host’s response to infection [99] (Table 2).

### 4.3. Amelioration of Intestinal Inflammation

**Figure 3:**
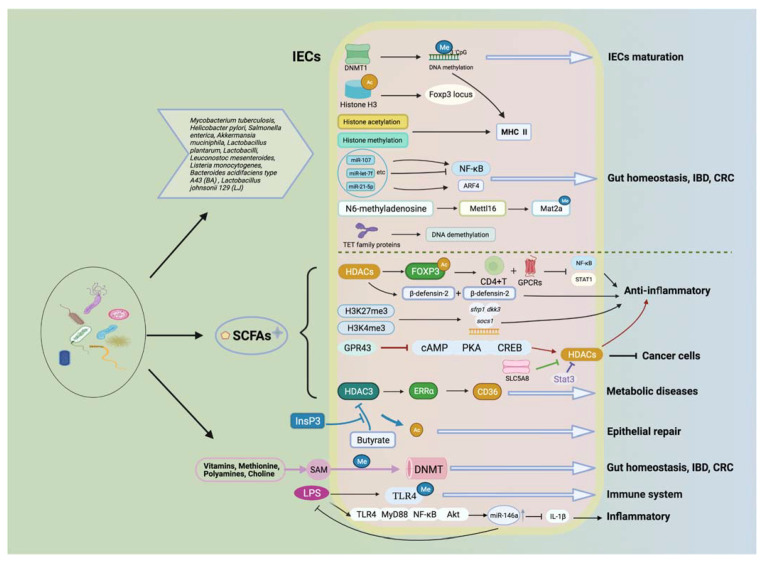
Gut microbiota and its metabolites affect health and diseases via multiple epigenetic pathways. Gut microbiota along with its metabolites (e.g., SCFAs) are involved in health and disease through multiple epigenetic mechanisms, including affecting transporter activities (e.g., DNMTs, HMT, HAT, and HDACs), providing methyl donors to participate in DNA methylation and histone modifications, and miRNAs that can lead to gene transcriptional modifications. These mechanisms can participate in a variety of biological processes, such as the maturation of IECs, the maintenance of intestinal homeostasis, inflammatory response, the development of metabolic disorders, and the prevention of colon cancer.

IBD can cause many health-associated problems and weaken the quality of life considerably. Gut microbiota, along with their metabolites, are intimately involved with the occurrence and development of IBD (Figure 3). The human diet digests by enzymatic hydrolysis to produce indigestible resistant starch (RS), which is the substrate for gut microbiota metabolism that has been demonstrated to reduce intestinal inflammation [144]. The effect of RS-4-derived butyrate on epigenetic suppression of proinflammatory genes has been investigated in vivo and in vitro. Mice in the RS 4 group displayed higher cecal butyrate content and an increased degree of trimethylation of histone 3 (H3K27me3) lysine 27 of the NFκB1 promoter in colon tissue compared to the control. In vitro, H3K27me3 enrichment was inversely correlated with concentration-dependent downregulation of NFκB1 in sodium-butyrate-treated human colonic epithelial cells [119]. Another gut microbiota metabolite—choline—can provide methyl donors for DNA methylation, which can be derived from betaine, choline, vitamin B12, and folate. According to [57], supplementation of choline as a methyl donor can adjust the microbiota composition and change the offspring’s colitis susceptibility in maternal mice.

It has been shown that microbiota could modulate the expression patterns of host miRNA [145]. Gut microbiota downregulates the expression of miR-107 in dendritic cells and macrophages, affecting the activity of the MyD88 and NF-κB pathways, and subsequently impacting immune homeostasis and the expression of target gene IL-23p19 [94]. Studies have found that infection with *Mycobacterium tuberculosis, Helicobacter pylori,* and *Salmonella enterica* can change the miRNA sequence in cells [95,96] (Table 2); *Mycobacterium tuberculosis* downregulates miR-let-7f by secreting ESAT-6, and Mir-let-7f targets TNFAIP3, a negative regulator of the NF-κB pathway that activates host immune responses and reduces microbial survival [146]. This evidence provides new approaches for the treatment of IBD and the maintenance of a host’s intestinal homeostasis.

Furthermore, a variety of metabolites derived from gut microbiota can also modulate intestinal inflammation via epigenetic pathways. As has been shown, SCFAs exert an anti-inflammatory effect by inhibiting histone deacetylases and specific ligands for G-protein-coupled receptors (GPCRs) [104]. Recent studies have revealed that gut microbiota can control the differentiation of T cells through histone modification, and then affect the intestinal inflammatory state. Almost all Treg cells are produced in the thymus (tTreg), and partly in the peripheral (pTregs), expressing the transcription factor Foxp3 and playing a vital role in the inhibition of intestinal inflammation [147]. SCFAs play an anti-inflammatory role as HDAC inhibitors by stimulating histone acetylation, increasing FOXP3 protein acetylation and gene expression in CD4+T cells, and promoting the differentiation of Treg cells [105,106,107]. The inhibition of HDAC by SCFAs promotes the expression of β-defensin-2 and β-defensin-3, which eventually leads to protection against severe infections [108]. On the other hand, macrophages can clear invading pathogens, regulate the inflammatory response, and maintain intestinal homeostasis. When gut microbiota are imbalanced or the host’s defense against invading microbiota is out of control, intestinal inflammation may occur. An experiment by Schulthess et al. showed that butyrate, a metabolite of gut microbiota, stimulates the differentiation of monocytes into macrophages by inhibiting histone deacetylase 3 (HDAC3), which induces the antimicrobial activity of intestinal macrophages and enhances intestinal resistance to pathogens [115]. HDAC1 and HDAC2 are important histone deacetylase epigenetic regulators. The deletion of *Hdac1* and *Hdac2* in IECs may induce dysbiosis of the mucosal barrier, thus causing chronic inflammation; the JAK/STAT pathway has been revealed to be a key mediator in this bioprocess [148].

However, the mechanism of microbial metabolites maintaining intestinal epithelial homeostasis and promoting epithelial repair function is affected by many factors. Here, we summarize the relevant mechanisms to provide research ideas for subsequent studies. Zheng et al. found that SCFAs could promote the formation of the epithelial barrier through an IL-10RA-dependent mechanism. SCFAs (especially butyrate) can promote the formation of the IEC barrier and induce IL-10RA mRNA, IL-10RA protein transactivation by activating STAT3 and inhibiting HDAC in human IECs. The loss of or an increase in IL-10RA expression is directly related to the formation of the IEC barrier; butyrate inhibits the permeability of the epithelial barrier through an IL-10RA-dependent mechanism and then promotes the expression of the tight junction protein claudin-2 [114] (Table 3). Therefore, microbial-derived butyrate can inhibit claudin-2 and promote the formation of the intestinal epithelial barrier through an IL-10RA-dependent mechanism. Wu et al. found that butyrate, an SCFA extracted from gut microbial populations, inhibited HDAC activity in vitro. Meanwhile, colonizing butyrate-producing microbiota (*Faecalibacterium prausnitzii*) in GF mice could significantly inhibit the activity of HDAC in IECs. HDAC activity was significantly increased in mice with intact gut microbiota, demonstrating the interaction between gut microbiota and HDACs. Meanwhile, *E. coli* can produce phytate-metabolizing phytase, which antagonizes the inhibitory effect of butyrate on intestinal HDAC3 by metabolizing phytate and producing inositol-1,4,5-triphosphate (InsP3), as well as inducing HDAC3 activation, promoting intestinal epithelial cell proliferation, repairing intestinal injury, and improving intestinal function. It was further verified that by supplementing mice with DSS-induced UC with InsP3, the survival rate of mice rose notably, showing phenotypic characteristics of reduced inflammation and epithelial regeneration [52].

Butyrate and propionate produced by gut microbiota can affect the differentiation or function of T cells, macrophages, and dendritic cells in the host. Recent studies have found that low-dose SCFAs can directly affect the intrinsic function of B cells and increase DNA recombination (CSR) moderately. High-dose SCFAs can reduce the expression of AID and Blimp1, CSR, somatic hypermutation, and plasma-cell differentiation in a wide range of physiologies. In human and mouse B cells, butyrate and propionate regulate *Aicda* and *Prdm1* mRNA-3′UTRs by inhibiting HDAC of these genes. SCFAs, as HDAC inhibitors, impair intestinal and systemic T-dependent and T-independent antibody responses, and inhibit the production of autoantibodies and autoimmunity in mice with lupus erythematosus [121].

### 4.4. Mitigation of Metabolic Diseases

Through epigenetic approaches, gut microbiota and their metabolites can affect the development of metabolic disease, and they show promising values in clinical treatment (Figure 3). For example, butyrate weakens the progression of nephropathy, in part via DNA methylation and histone deacetylase (HDAC)-dependent programs to protect podocytes, which involves the activation of necessary genes for podocyte function and GPR109A [149]. High levels of polyamines and choline present in the gut may come from the diet or be produced by the host or gut microbiota; these are essential nutrients for human life and are important methyl donors in epigenetics [150]. Metabolomic studies in mice have shown that levels of putrescine and spermidine (not spermine) in the gut are mainly dependent on colonic microbiota [151]. Supplementation with exogenous polyamines reverses the overall aberrations in DNA methylation associated with aging, and these aberrant DNA methylation profiles are also associated with metabolic disorders [125,126,127]. Romano et al. verified that choline-utilizing microbiota compete with the host, significantly affecting the levels of methyl donor metabolites in plasma and liver and exhibiting the biochemical features of choline deficiency. At the same time, mice on a diet carrying high levels of choline-depleting microbiota are more sensitive to metabolic diseases [130]. Gut microbiota can also induce demethylation by oxidating 5mC to 5hmC via ten-eleven translocation (TET) family proteins. *Mycobacterium tuberculosis* (*M. tuberculosis*) was used to infect human dendritic cells (DC) to induce DNA demethylation of enhancer elements at thousands of distal sites. A comprehensive analysis showed that these loci were enriched in the regions of immune transcription factors, active histone marker sites, and increased chromatin accessibility, which strongly correlated with gene expressions [97]. The production process of tricarboxylic acid (TCA) is affected by acetyl-CoA produced by SCFAs; among these intermediates, alpha-Ketoglutarate plays a role in the cosubstrate of ten-eleven translocation (TET) dioxygenases to control the demethylation processes, while TET enzymes are inhibited by fumarate and succinate, thereby increasing DNA methylation levels [14]. Recently, it was found that when different Bacteroidetes:Firmicutes ratios were present in the gut microbiota composition of blood and adipose tissue of obese subjects, DNA methylation patterns and genome-wide DNA methylation profiles were completely different in feces. There are 258 differentially methylated genes, such as HDAC7 and IGF2BP2, all of which are associated with glucose and energy homeostasis [152]. These data suggest that the changes caused by the stimulation of gut microbiota can affect the location of 5mC and 5hmc in the host, resulting in epigenetic changes.

Gut microbiota are involved in metabolic syndrome through pathways of lipid and glucose metabolism, satiety, and chronic low-grade inflammation. As fermentation products, SCFAs can participate in metabolism-related epigenetic regulation through free-fatty-acid receptors (FFARs) and other short-chain fatty-acid receptors. Through the use of GLP-1 receptor agonist intervention in obese and type 2 diabetic patients, studies have found a potential interplay between gut microbiota and epigenetics compared with lean controls. The *Butyryl-CoA: Acetate-CoA*-transferase gene can be used as a marker of butyrate production. In contrast to the *butyryl-CoA: Acetate-CoA*-transferase gene, it is significantly lower in type 2 diabetic patients with both the *Clostridium cluster IV* and the *Clostridium cluster XIVa*. During the intervention period, methylation of five CpGs in the FFAR3 promoter region was significantly lower in obese and type 2 diabetic patients, as body mass index increased in the former. These results indicate a significant association between FFAR3 hypomethylation and increased body mass index in patients with metabolic diseases. LINE-1 is often highly methylated; maintaining DNA stability and its mutants can be associated with cancer, age, ischemic heart disease, stroke, plasma fasting glucose, and lipid levels. However, LINE-1 showed hypomethylation in the obese group compared with the lean control group. Therefore, it has been indicated that an altered gut microbiota structure and metabolites can affect the epigenetic regulation of genes in obesity and type 2 diabetes [118].

Gut microbiota can regulate proteins in the intestinal epithelium by producing metabolites, which affect the metabolic phenotype of the host. Whitt et al. hypothesized that in IECs, microbial-derived butyrate integration with HDAC3 can maintain intestinal homeostasis. The administration of HFD to control mice increased body weight, obesity, serum insulin, and decreased glucose tolerance, whereas HDAC3^ΔIEC^ mice fed by HFD did not develop obesity and showed reduced serum triglyceride levels, less hepatic fat, and smaller adipocytes compared to HFD-fed control mice. Compared with normal mice, IEC mice for HDAC3 altered the expression levels of genes that regulate metabolism in response to microbial populations (e.g., *Chka*, *Mttp*, *Apoa1*, and *Pck1*) and accumulated glycerolipid. At the same time, microbiota-derived butyrate was reduced in obese mice. Compared with HDAC3^ΔIEC^, the activity of HDAC3 was significantly reduced and it increased the expression of *Pck1*, leading to significant weight loss after butyrate treatment in control mice. However, the disruption of HDAC3 in IECs of obese mice resulted in weight loss and an improved metabolic status [116]. After combining these pieces of evidence, it should be clear that butyrate could inhibit HDAC3 in IECs, and thereby reduced the activity of diet-induced obesity.

In relation to the circadian clock, Kuang et al. reported that the gut microbiota programs diurnal metabolic rhythms through HDAC3 in mice. The circadian rhythm may regulate daily oscillations in histone acetylation, expression of metabolic-related genes, and nutrient uptake. HDAC3 also synergistically activates estrogen-related receptor α (ERRα), induces microbiota-dependent rhythmic transcription of the lipid transporter gene CD36, and promotes lipid absorption and diet-induced obesity [93] (Table 3).

### 4.5. Promotion of Tumor Suppression

Intestinal microflora and their related metabolites have been proven to have valuable tumor-suppression bioactivities (Figure 3). Previous studies have shown that a fiber-rich diet can induce strong tumor inhibition in a microbiota metabolite butyrate-dependent manner. Dietary supplementation with foods such as catechins and black raspberries helps to provide methyl donors or alter DNMTs activity to suppress tumor cells. The methylation phenotype of CpG island is characterized by the DNA hypermethylation of the promoters of several repressed genes implicated in the inactivation of various pathways associated with tumorigenesis [128]. However, microbial degradation of catechins (mainly epigallocatechin-3-gallate (EGCG)) can inhibit the activity of DNMTs, mostly by decomposing o-heterocycles and dihydroxylation to produce phenolic acids, which can be metabolized by *Bacteroides eggerthi* to benefit human health [128,153]. Meanwhile, supplementation with EGCG can reduce the Firmicutes:Bacteroidetes ratio, which is manifested by an increase in DNMT1 in the colon [129]. Black raspberries increase the abundance of butyrate-producing microbiota (such as Anaerostipes) and anti-inflammatory microbiota (such as Akkermansia and Desulfovibrio); moreover, the metabolism of microbiota leads to a reduction in DNMT1 and methylation levels of genes involved in the Wnt signaling pathway in tumor tissues [154].

MiRNAs are considered to be important regulators of cancer cell homeostasis, and there is a growing body of evidence demonstrating their compelling role in tumor suppression and apoptotic pathways [117]. Meanwhile, butyrate is considered to play an important role in inducing apoptosis in tumor cells. Butyrate promotes cell proliferation by serving as a carbon donor for acetyl-CoA and histone acetylation in normal colonocytes. In these conditions, low amounts of butyrate favor HAT activity and proliferation; however, high dosage promotes HDAC inhibition and apoptosis [155,156]. Butyrate is metabolized less in tumors and plays its role as a histone deacetylase inhibitor because of the Warburg effect, thus stimulating histone acetylation and affecting cell apoptosis and proliferation [157]. Hu et al. found that butyrate seems to have a tumor-inhibitory effect, and can suppress the proliferation and induce apoptosis of colon cancer cells by inhibiting miR-92a transcription [120] (Table 3). The expression level of miR-92a in sporadic colorectal cancer tissues is seven times that of adjacent normal tissues, and a supplement of butyrate in human cancer cell lines can lower the levels of miR-17-92a and miR-92a. This discovery is related to the inhibition of key Myc oncogene and the enhancement of CDKN1C (also known as p57) expression, which leads to proliferation inhibition and the apoptosis of cancer cells. For instance, in a randomized controlled trial, healthy volunteers were given a lump of high red meat (HRM) (300 g/day) or high red meat plus a resistant fiber supplement for four weeks. Results showed that HRM diet increased the expression of cancer-causing miR-17-92 clusters and cell proliferation in the rectal mucosa, while additional fiber supplementation could increase butyrate production, reduce the risk of HRM, and restore the baseline level. These relationships reflect that the colonization of dietary-derived fiber or butyrate-producing microbiota in the gut may prevent colorectal cancer [158].

The failure of chemotherapy induces recurrence and poor prognosis in CRC. To investigate whether gut microbiota play a role in chemoresistance in CRC patients, Yu et al. found that reducing specific gut microbiota in the gut of CRC patients improved their response to chemotherapy and reduced cancer recurrence rates. Further studies revealed that providing *Fusobacterium nucleatum* to patients after chemotherapy could effectively help them reduce chemotherapy resistance by coordinating Toll-like receptors, miRNAs (miR-4802, etc.), and autophagic networks. Therefore, the detection and targeted therapy offered by *F. nucleatum* are both helpful for the prognosis and treatment of patients [102] (Table 2).

Probiotic supplementation can prevent and treat colon cancer by regulating miRNAs. *Leuconostoc mesenteroides* is a probiotic used in dairy products, and HT-29 is a human colon cancer cell line. Coculture of *L. mesenteroides* with HT-29 cells showed that *L. mesenteroides* could downregulate miRNA-21 and miRNA-200b effectively, and then promote the apoptosis of colon cancer cells. The results imply that probiotics play an important role in mediating key carcinogenic miRNAs, and can serve as complementary and alternative pathways for colon cancer therapy [100]. However, its intrinsic mechanism needs to be explored in further studies.

Butyrate, a fermentation product of gut microbiota, is a substrate of SLC5A8 and an agonist of GPR109A. Compared with normal mice, levels of SLC5A8 mRNA and GPR109A mRNA in the colon and ileum of GF mice were significantly decreased. When these GF mice were maintained under conventional conditions for 3–4 weeks, the expression of SLC5A8 and GPR109A changed, at both the mRNA and protein levels. Therefore, gut microbiota regulate SLC5A8 and GPR109A expression in the colon [159]. SLC5A8 is a plasma membrane transporter and a Na^+^-coupled transporter of many SCFAs, including butyrate [110,111] (Table 3). In colon cancer cells, the gene *slc5a8* was found to be silenced via DNA methylation modifications; when re-expressed, *slc5a8* can cause tumor cell growth arrest and apoptosis [160]. Studies revealed that tumor cells can inhibit the expression of *slc5a8* on the apical membrane of colonocytes by preventing butyrate from entering cells. As with SLC5A8, GPR109A may also undergo DNA methylation in colon cancer, but GPR109A can be re-expressed in colon cancer cells in the presence of butyrate, leading to the death of tumor cells [161].

GPR43 (free-fatty-acid receptor 2; FFAR2) is a cell-surface G-protein-coupled receptor. Activated by SCFAs (mainly acetate and propionate precursors) in the colon, GPR43 can promote the accumulation of colon Treg cells, thus inhibiting inflammation and promoting intestinal microflora homeostasis [162,163,164]. GPR43^−/−^ mice were shown to be more susceptible to inflammation in the experimental colitis model [162,165]. In colon cancer, the expression of GPR43 was significantly decreased [166]. Based on this point, it has been supposed that GPR43 may play a role in the prevention of tumorigenesis [109]. The mouse model of inflammation-promoting carcinogenesis in the colon could be induced by *Apc^Min/+^* and azomethane (AOM), and studies showed that the GPR43^−/−^ genotype promoted the development of colonic adenoma in *Apc^Min/+^*/DSS and AOM/DSS mice. Moreover, the downstream cAMP-PKA (protein kinase A)-CREB (cAMP responsive element-binding protein) pathway was enhanced, resulting in the overexpression of HDACs. Moreover, butyrate needs the involvement of GPR43 to inhibit HDAC expression. The above evidence suggests that GPR43 plays a vital role in the suppression of colon cancer, and that it does so in a similar manner to GPR109A.

## 5. Conclusions

The interplay between gut microbiota and epigenetic mechanisms is of great significance for disease prevention and for unveiling the truth of disease. The human body and gut microbiota constitute a symbiotic community that changes dynamically with the influence of the living environment, eating habits, and other acquired factors. Host epigenetics will respond to these stimuli and then produce bioactive compounds to regulate the diversity and abundance of intestinal microflora. Several recent studies have shown that gut microbiota can utilize nutrients like dietary fiber to produce SCFAs, which have comprehensive effects on the human immune system and other physiological processes. SCFAs and other microbiota-derived metabolites could reprogram the transcriptome of the host through epigenetic mechanisms of DNA methylation and histone modifications.

In the present work, we summarized the interplay mechanism between gut microbiota and various epigenetic factors (DNA methylation, histone modification, etc.) and how their mutual functions affect health and disease. This interplay can exert a wide range of regulatory effects on the human body, especially in the maintenance of intestinal microecological homeostasis, repair of intestinal inflammation, treatment of metabolic disorders, and suppression of colon cancer. In maintaining intestinal balance, the interaction between host miRNAs and specific microbiota plays an important role. Research progress on the epigenetics–microbiota interplay sheds new light on the treatment of UC, which cannot be cured satisfactorily nowadays due to our limited understanding of its etiology and pathogenesis.

In addition, the interaction between epigenetics and intestinal microflora also plays a crucial role in the progress of metabolic diseases. For instance, gut microbiota could utilize HDAC3 to program diurnal metabolic rhythms to regulate the oscillations in histone acetylation, metabolic gene expression, and nutrient uptake; meanwhile, HDAC3 can induce microbiota-dependent rhythmic transcription of the lipid transporter gene CD36 and diet-induced obesity. Moreover, in terms of cancer prevention and treatment, we discussed the fact that probiotics play an important role in mediating key oncogenic miRNAs, and they can be used as a complementary approach for colon cancer treatment. Furthermore, butyrate, a microbial metabolite, appears to have a tumor-suppressive function by inhibiting miR-92a transcription.

In summary, all of the above evidence indicates the significance of the interplay between epigenetics and gut microbiota. This interaction plays a multifaceted role in health maintenance and disease prevention. Further exploration is still needed to explore the deep mechanism behind this interaction to enrich our understanding and promote its application in more fields.

## Data Availability

Not applicable.

## Abbreviations

SCFAs	short-chain fatty acids
IECs	intestinal epithelial cells
IBD	inflammatory bowel disease
InsP3	inositol-1,4,5-triphosphate
DHPP	methyl-donating folate from pteridine precursors
pABA	p-aminobenzoic acids
LPS	lipopolysaccharide
SAM	S-adenosylmethionine
DNMT	DNA methyltransferase
